# Eco-Friendly Hydrogels from Natural Gums and Cellulose Citrate: Formulations and Properties

**DOI:** 10.3390/gels11121005

**Published:** 2025-12-12

**Authors:** Giuseppina Anna Corrente, Fabian Ernesto Arias Arias, Eugenia Giorno, Paolino Caputo, Nicolas Godbert, Cesare Oliviero Rossi, Iolinda Aiello, Candida Milone, Amerigo Beneduci

**Affiliations:** 1 Dipartimento di Ingegneria, Università di Messina, Contrada di Dio, 98166 Messina, ME, Italy; 2 Facultad de Ciencias, Escuela Superior Politécnica de Chimborazo (ESPOCH), Riobamba 060155, Ecuador; 3 PC-SMARTech Laboratory, Dipartimento di Chimica e Tecnologie Chimiche, Università della Calabria, 87036 Arcavacata di Rende, CS, Italy; 4 MAT_InLAB (Laboratorio di Materiali Molecolari Inorganici), Dipartimento di Chimica e Tecnologie Chimiche, Università della Calabria, 87036 Arcavacata di Rende, CS, Italy; 5 PCAM LAB, Dipartimento di Chimica e Tecnologie Chimiche, Università della Calabria, 87036 Arcavacata di Rende, CS, Italy; 6 CNR-Nanotec, UoS di Rende, Università della Calabria, 87036 Arcavacata di Rende, CS, Italy; 7 LPM-Laboratorio Preparazione Materiali, STAR-Lab, Università della Calabria, Via Tito Flavio, 87036 Arcavacata di Rende, CS, Italy

**Keywords:** hydrogels, natural gums, cellulose citrate, thermal properties, rheological properties

## Abstract

The design of sustainable hydrogel materials with tunable mechanical and thermal properties is essential for emerging applications in flexible and wearable electronics. In this study, hydrogels based on natural gums such as Guar, Tara, and Xanthan and their composites with Cellulose Citrate were developed through a mild physical crosslinking process, ensuring environmental compatibility and structural integrity. The effect of cellulose citrate pretreatment under different alkaline conditions (0.04%, 5%, and 10% NaOH) was systematically investigated using Fourier Transform Infrared Spectroscopy (FT-IR), Thermogravimetric Analysis (TGA), and dynamic rheology. Overall, the results show that the composites exhibit different properties of the hydrogel networks compared to the pure hydrogel gums, strongly depending on the alkaline treatment. In all composite hydrogels, a significant increase in the number of interacting rheological units occurs, though the strength of the interactions decreases in Guar and Tara composites, which exhibit partial structural destabilization. In contrast, Xanthan–Cellulose Citrate hydrogels display enhanced strong gel character, and crosslinking density. These improvements reflect stronger intermolecular associations and a more compact polymer network, due to the favorable H-bonding and ionic interactions among Xanthan, Cellulose and Citrate mediated by water and sodium ions. Overall, the results demonstrate that Xanthan–Cellulose Citrate systems represent a new class of eco-friendly, mechanically robust hydrogels with controllable viscoelastic and thermal responses, features highly relevant for the next generation of flexible, self-supporting, and responsive soft materials suitable for wearable and stretchable electronic devices.

## 1. Introduction

Hydrogels are three-dimensional polymeric networks capable of absorbing and retaining large amounts of water or biological fluids, owing to their hydrophilic functional groups and network architecture [1,2,3]. These materials exhibit unique swelling behavior, often increasing their volume and mass by several orders of magnitude relative to their dry state [4]. The water absorption capacity of hydrogels is governed by various molecular interactions, including hydrogen bonding, capillary forces, osmotic pressure, and electrostatic interactions, all of which contribute to their physicochemical properties and functionality. They are broadly classified as physical or chemical gels, depending on the type of crosslinking within the polymeric matrix [5,6,7]. Physical hydrogels are stabilized by reversible interactions such as hydrogen bonds or entanglements, making them sensitive to environmental conditions like pH and temperature [8]. In contrast, chemical hydrogels possess covalent crosslinks, providing higher mechanical stability but reducing reversibility and biodegradability [1,6].

**Figure 1:**
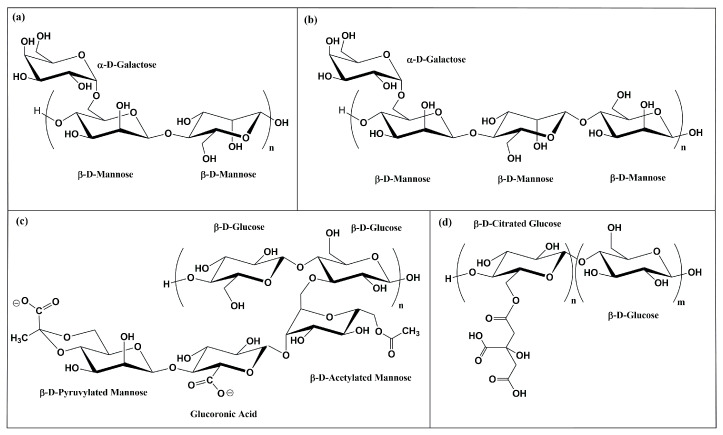
(**a**) GG consists of D-mannose and D-galactose in a 2:1 ratio; (**b**) TG consists of D-mannose and D-galactose in a 3:1 ratio; (**c**) XG is made of D-glucose units (β-1,4) with a C3 branch every two residues carrying a trisaccharide of two mannose units and one glucuronic acid; (**d**) CC derives from cellulose (β-D-glucopyranose, β-1,4), in which almost half of the primary hydroxyl groups are esterified with citric acid.

In recent years, the development of eco-friendly and biodegradable hydrogels has gained considerable attention due to growing environmental concerns and the need for sustainable alternatives to synthetic polymers, such as polyacrylate-based hydrogels, which pose significant ecological challenges [4]. In this context, natural polysaccharides, especially those derived from plant exudates known as natural gums, have emerged as promising raw materials for hydrogel formulation [2,8,9,10,11]. These gum-based hydrogels offer several advantages, including biocompatibility, biodegradability, renewability, and the ability to form hydrophilic networks with tunable mechanical and rheological properties. Among them, Guar gum (GG) and Tara gum (TG) of vegetal origin, and Xanthan gum (XG) of bacterial origin, have attracted considerable interest due to their availability and structural diversity (Figure 1a–c), which enables them to gel, thicken, or act as stabilizers of emulsions and suspensions, resulting in distinct rheological properties and a broad range of applications [9]. GG is a galactomannan with high viscosity and thickening ability, commonly used in food and pharmaceutical applications [12]. TG, also a galactomannan, differs from GG for the ratio between Galactose and Mannose units, 1:2 in the case of GG and only 1:3 in the case of TG. This lower content in branched Galactose units in TG compared to GG confers the gum’s intermediate properties with a moderate viscosity, a lower hydration rate but, overall, a better thermal and acid stability than GG, reason for its use as biodegradable films and controlled release systems [13]. Instead, XG is an anionic polysaccharide owing to the presence of glucuronic acid and pyruvate residues. XG exhibits excellent shear-thinning behavior and stability under various conditions in particular across wide pH range values (pH = 1 to 13) [14].

Moreover, cellulose, the most abundant biopolymer on Earth, has been increasingly studied for its potential in hydrogel synthesis, particularly in its chemically modified forms [15,16]. Cellulose, a linear polymer of 1,4-β-D-Glucose units, is insoluble in water and therefore often requires chemical functionalizations to be successfully employed. One of its derivative, Cellulose Citrate (CC) (Figure 1d), is obtained through esterification of Cellulose with citric acid, introducing carboxyl functional groups into the polymer backbone, which enhance its water affinity, swelling behavior, and the potential for crosslinking via hydrogen bonding and/or ionic interactions [17].

The integration of natural gums with modified cellulose derivatives offers a versatile and sustainable platform for the design of smart hydrogels with tunable mechanical, thermal and responsive properties, specifically targeting flexible and wearable electronic applications. Recent research on cellulose-based conductive hydrogels highlights their suitability for flexible sensors and wearable energy-storage devices, thanks to attributes such as stretchability, ambient stability and multifunctionality [18,19]. Moreover, bio-based hydrogels derived from polysaccharides are increasingly explored as eco-friendly alternatives to petrochemical polymers in stretchable electronics, where they can serve as dielectric layers, stretchable conductors or self-healing skins [20,21]. Cellulose citrate (CC) offers several advantages over other cellulose derivatives, particularly for biocompatible, sustainable materials with good adsorption or composite performance [22,23]. Unlike simple cellulose esters (acetate, propionate, butyrate), which mainly improve solubility and thermoplasticity, CC introduces multiple carboxyl groups per monomer, giving a stronger anionic character, higher hydrophilicity, and enhanced ability to adsorb cationic dyes, metals, and proteins [22,23]. Compared with cellulose nitrate, CC is much safer and greener, as cellulose nitrate is highly flammable and requires hazardous nitration reagents [22,23]. Relative to cellulose ethers (CMC, HEC, HPC, MC, EC), CC also enables covalent crosslinking via citrate ester bridges [22,23]. Unlike cellulose succinates or maleates, CC provides more acidic groups per repeat unit and offers intra- and inter-chain crosslinking through citric acid’s three carboxyl and one hydroxyl groups [22,23]. Moreover, CC can be prepared by a “green” route from cellulose and citric acid under mild conditions, avoiding harsh reagents [17]. Its combination of a robust cellulose backbone and highly functional citrate groups makes CC a promising bio-derived filler or reinforcing phase in polymer composites, with reported toughness enhancements via hydrogen-bond networks [24]. In this context, the combination of natural gums and cellulose citrate presents an under-exploited route to achieve biopolymeric gel networks that meet the mechanical durability and flexibility demands of next-generation soft electronic devices.

In this study, we report the formulation and a comprehensive characterization of hydrogels based on GG, TG, and XG, as well as composite hydrogels incorporating CC, with the aim of investigating their thermal behavior, structural features, and rheological properties. CC was chosen because its pendant citrate groups promote crosslinking/gelation [25,26], while its cellulose backbone enhances the mechanical performance of gum-based hydrogels. For this study, CC was synthesized according to the reported procedure [17] which allowed us to introduce 46% of citrated units into the polymer backbone. Due to this relatively high content of carboxylic groups, before incorporation into the natural gums, CC was pretreated with sodium hydroxide to investigate its role in the composite gels.

The alkaline pretreatment serves three main purposes:

(i.)   Solubilization: CC was dissolved in water to ensure homogeneous mixing with the gums. [27].
(ii.)  Network tuning: through controlled hydrolysis, which generates oligomers of varying chain lengths and partial in situ release of citrate that modulate the gel structure upon mixing with the gums [28,29].
(iii.) Biostability: to suppress microbial growth (e.g., yeast), allowing storage of the hydrogels for months, even at room temperature.

**Figure 2:**
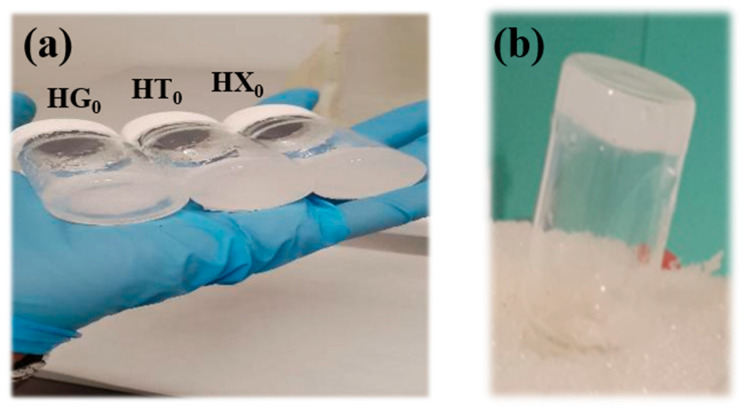
Hydrogels obtained from natural gums: (**a**) photographs of the prepared hydrogels; (**b**) inverted vial of a representative sample demonstrating the formation of stable, self-supporting gels.

Therefore, studying the effect of the alkaline pretreatment, in terms of the amount of NaOH added to CC, is a key step in hydrogel formulation. To this end, we screened various formulations differing for the ratio between the components and as a function of the NaOH concentration used for CC pretreatment. The resulting materials were characterized by Fourier Transform Infrared Spectroscopy (FT-IR) for structural analysis, Thermogravimetric Analysis (TGA) for thermal characterization, and rheological measurements to evaluate their viscoelastic performance. All natural gums investigated were able to form hydrogels (Figure 2) exhibiting a strong-gel character. The incorporation of CC affected the gel properties differently depending on the gum type: a similar trend was observed for TG and GG, while XG displayed a distinct response. Moreover, these effects were found to depend on the alkaline pretreatment applied to CC, emphasizing the critical role of the chemical environment in tailoring the structural and rheological properties of the resulting hydrogels.

## 2. Results and Discussion

### 2.1. FT-IR Spectral Analysis of the Hydrogels

**Figure 3:**
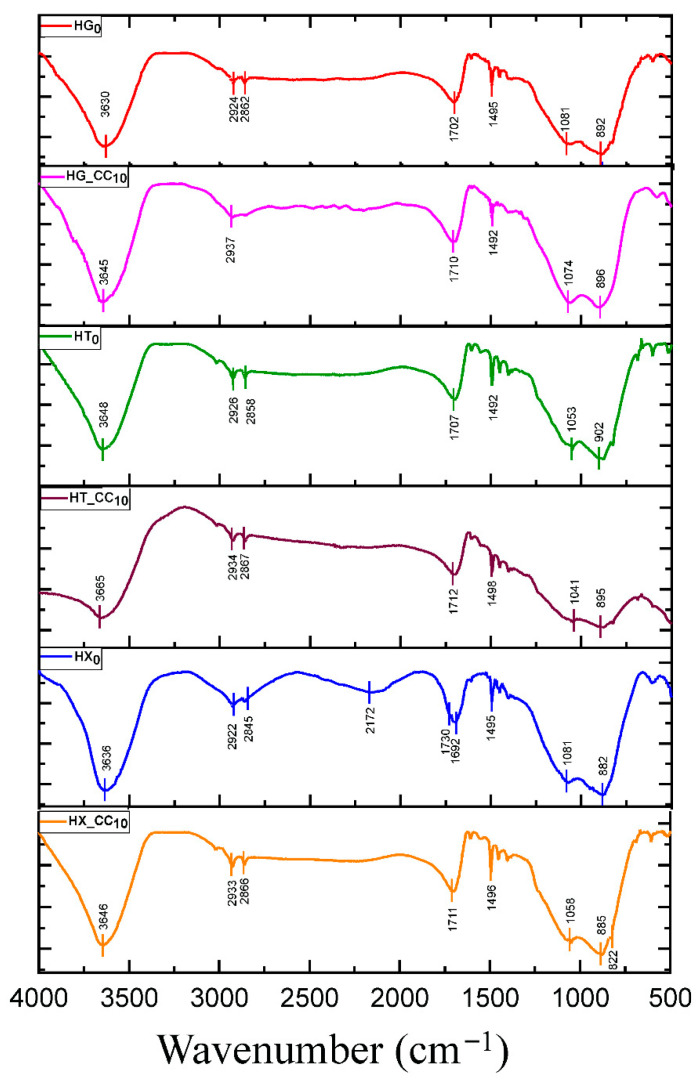
Comparison of FT-IR spectra of pure gum hydrogels (HG_0_, HT_0_, HX_0_) and that of the corresponding composites.

The FT-IR spectra of the pure gum hydrogels, reported in Figure 3, are highly similar to one another and exhibit the characteristic absorption bands of their polysaccharide networks, consistent with those observed in the corresponding gum powders and previously reported in [30]. In the powder samples, the band at ~1650 cm^−1^ is attributed to the bending vibration of bound water [31,32]. In the hydrogels, however, this band appears broader and slightly shifted toward higher wavenumbers (~1700 cm^−1^). This broadening and shift suggest a stronger structuring of water molecules within the gel network. The more rigid hydrogen-bond network in the hydrated state likely causes the OH bending to occur over a wider range of energies. In the case of xanthan hydrogel, this intense and broadened OH bending band partially overlaps with the C=O stretching vibration around 1730 cm^−1^, making the latter scarcely distinguishable in the spectrum. Figure 3 and Figure S1 also show the FT-IR spectra of the hydrogel–CC composites, which are essentially governed by the pristine hydrogels, with only minor contributions from CC (Figure S2) [17]. In particular, the characteristic ester carbonyl band of CC at 1736 cm^−1^ is barely discernible, suggesting that the polysaccharide framework largely masks its signal. This behavior is consistent for both galactomannan and glucomannan-based hydrogels, whose spectra remain nearly indistinguishable from those of the neat gels.

### 2.2. Thermogravimetric Analysis (TGA)

#### 2.2.1. Pure Gum Hydrogels (HG, HT, and HX)

**Figure 4:**
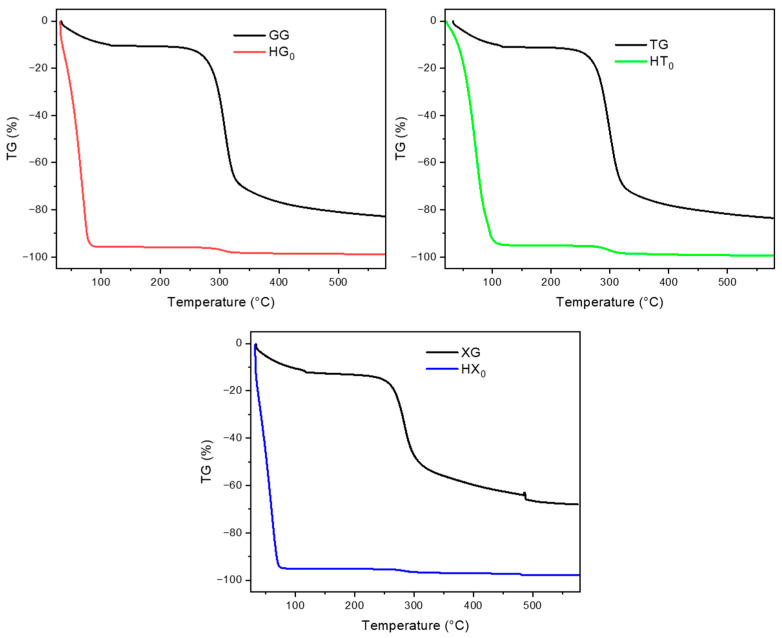
Comparison of TGA curves of natural gums in powder form and their corresponding hydrogels.

The thermal stability of hydrogels was investigated by TGA. As shown in Figure 4, all pure gum hydrogels exhibit a characteristic two-step weight loss profile. The first step occurs below 100 °C, corresponding to the evaporation of free water and that of the physically entrapped water molecules within the polymer matrix. This initial mass loss reflects the high water content typical of hydrogels, in which water is retained within the three-dimensional polymeric network. The second stage, a more gradual and less intense mass loss occurring at about 280–300 °C, is attributed to the thermal decomposition of the polysaccharide backbone. This includes the cleavage of glycosidic linkages and degradation of side chains. However, due to the high water content and the relatively low solid fraction of the hydrogels, the extent of polymer decomposition is minimal, resulting in a smaller total weight loss in this region. Compared to the TGA of the corresponding gums in powder (Figure 4), the hydrogel samples exhibit a markedly different thermal profile in the early stage of the TGA curve. In particular, where the hydrogels display a more pronounced initial mass loss below 100 °C, due to their high water content, the dry powders show minimal weight change in this region, confirming the absence of free water and highlighting the impact of hydration on the thermal behavior of the materials.

#### 2.2.2. Cellulose Citrate Powder

The TGA curve of CC (Figure S3) displays a two-step degradation profile. An initial gradual mass loss up to ~115 °C which corresponds to evaporation of adsorbed water, typical of hydrophilic polysaccharides. This is followed by a second major degradation occurring between 270 °C and 400 °C, corresponding to the thermal decomposition of the cellulose citrate which includes citrate functional groups decomposition, backbone depolymerization, dehydration, and formation of volatile degradation products. The broad temperature range and relatively early onset of degradation (~270 °C) suggest a moderate reduction in thermal stability compared to unmodified cellulose, possibly due to reduced crystallinity and the presence of ester bonds.

#### 2.2.3. Composite Hydrogels (HG_CC, HT_CC, and HX_CC Series)

**Figure 5:**
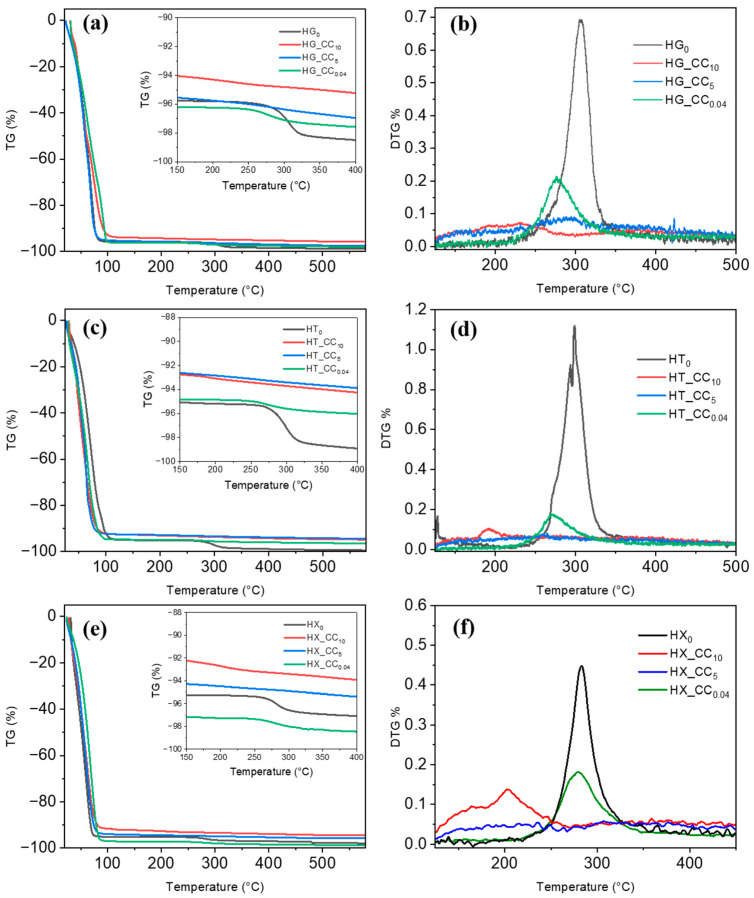
Comparison of TGA and DTG curves of pure hydrogels and that of the corresponding composites (**a**–**f**).

Figure 5 presents the thermal behavior of composite hydrogels incorporating CC prepared under different alkaline conditions (0.04%, 5%, and 10% NaOH), in comparison with the respective pure gum hydrogels. The composite hydrogels exhibited clearly distinct thermal profiles, with differences observed both in the low-temperature region (associated with water loss) and in the main degradation range (200–400 °C). In the low-temperature range (30–150 °C), the TGA traces show that the HG_CC and HX_CC composite hydrogels display a broader temperature interval for water loss than the corresponding pure gum hydrogels HG_0_ and HX_0_. This broadening of the dehydration interval, by approximately 30 °C, which is clearly visible in the DTG diagrams zoomed in the above T range (Figure S4a,c), suggests a more heterogeneous distribution and possibly stronger structuring of water within the gel matrix, reflecting a different polymer–water organization. Although the experiments were performed at a heating rate of 10 °C/min and are therefore not optimized for detailed kinetic analysis, these differences in the width and position of the water-loss step are systematic and reproducible over independent measurements. This behavior is qualitatively consistent with a higher degree of network structuring in HG_CC and HX_CC composite hydrogels, as also supported by the viscoelastic analysis presented in the Section 2.3. In contrast, the HT–CC hydrogels show a comparatively narrower dehydration range and an earlier onset of water loss with respect to the pure HT_0_ hydrogel (Figure 5c,d and Figure S4b), suggesting a less cohesive polymer–water network and a progressive weakening of its structural organization upon CC incorporation. These observations indicate that the alkaline environment significantly affects the hydration state and the way water is distributed and bound within the network, thereby allowing a partial tuning of the intermolecular organization during gel formation. It is also worth noting that the alkaline treatment controls the amount of free citrate anions formed in situ by partial CC hydrolysis, as well as the sodium ion concentration in the gel. Both factors can influence gelation and network structure. In particular, citrate species may participate in the gelation process through hydrogen bonding and electrostatic ion–ion interactions [33,34,35], involving sodium ions and the carboxylate groups of cellulose citrate (covalently bound) and those of XG located on glucuronic acid and pyruvylated mannose units.

In the high-temperature range, in the composite hydrogels prepared with the lowest NaOH concentration, a broad degradation process, extending from approximately 225 °C to 350 °C, is clearly visible. Interestingly, it is anticipated to a lower temperature with respect to either the degradation peak of the pure gum or that of CC, probably due to partial hydrolysis of the polymer chains. Hence, it is reasonable to assign this peak to the degradation processes of all polymers’ backbones. By contrast, at higher NaOH concentration, the composite hydrogels exhibit several consecutive low-intensity mass-loss events, consistent with partial alkaline hydrolysis of the polymer matrix already occurring during preparation. This produces oligomers of different chain lengths that decompose over a broader range of temperatures.

These results highlight the sensitivity of thermal behavior to both gum type and preparation method, with the alkaline strength playing a key role in modulating water interaction and possibly gel network structure.

### 2.3. Viscoelastic Properties

#### 2.3.1. Gum Hydrogels and Their Composites with Cellulose Citrate at 25 °C

**Figure 6:**
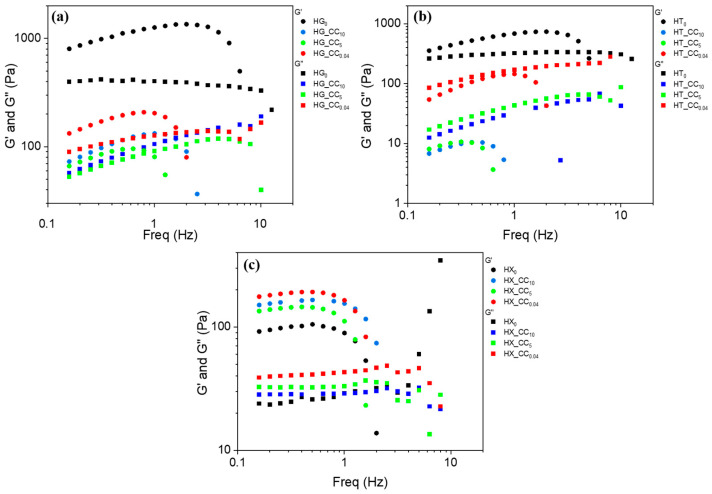
Comparison of the storage (G′) and loss (G″) moduli of pure gum hydrogels at 25 °C and of composite hydrogels as a function of NaOH treatment for (**a**) guar-, (**b**) tara-, and (**c**) xanthan-based systems. Measurements were performed in triplicate, and the reported data are representative of the average behavior and properties of the samples. The experimental variability across the three replicates is around 5%.

The dynamic rheological characterization of all pure gum hydrogels shows that, at 25 °C, all gums exhibit strong gel behavior, as indicated by the elastic modulus (G′) consistently exceeding the viscous modulus (G″) across all tested frequencies (Figure S5). This frequency-independent behavior is typical of the formation of highly connected and strongly interacting polymer networks [33]. A comparative analysis among the three systems revealed that the HG_0_ hydrogel exhibited the most structured gel-like character, with a G′ value reaching approximately 10^3^ Pa, which remained stable with increasing temperature. Upon incorporation of CC and exposure to alkaline conditions, both the magnitude of G′ and G″ and their frequency dependence are modified. These changes are likely to arise from a combination of effects, including (i) alterations in the molecular weight distribution and dispersity of the polysaccharides induced by NaOH treatment, (ii) the presence of low-molecular-weight citrate species that may act as plasticizers and modify the relaxation dynamics of the network, and (iii) changes in the water content and its distribution within the hydrogel, which can strongly affect viscoelastic properties. Specifically, when CC is incorporated into the HG hydrogels, both the elastic (G′) and viscous (G″) moduli decreased compared to the pure guar gel HG_0_, indicating a partial weakening of the polymer network. Nevertheless, all composite samples retained a clear solid-like behavior (G′ > G″) over the entire frequency range (Figure 6a). The relative magnitude of the moduli followed the trend HG_CC_0.04_ > HG_CC_10_ > HG_CC_5_, suggesting that the sample prepared with the lowest alkaline content (HG_CC_0.04_) preserved the gel structure most effectively, while the formulation obtained with 5% NaOH (HG_CC_5_) showed the greatest reduction in viscoelastic strength. This behavior indicates that the degree of alkalinity during CC preparation affects the structural organization of the guar–cellulose network in a non-linear manner: intermediate alkaline conditions appear to disrupt the network more markedly than either very mild or strongly basic environments. By contrast, the tara-based hydrogels showed an even greater sensitivity to CC addition. In these systems, the incorporation of cellulose citrate led to a complete loss of the gel-like behavior, irrespective of the alkaline pretreatment, with G″ exceeding G′ across the entire frequency range, indicating a transition from a solid-like to a predominantly viscous liquid response (Figure 6b). This pronounced destabilization suggests that the tara matrix is less compatible with cellulose citrate, and that the alkaline conditions used during preparation further disrupt the intermolecular associations necessary for gel network formation. This particular behavior may be attributed to the additional β-mannose unit of TG compared to GG. Indeed, the lower galactose/mannose ratio in TG generates linear mannose-rich domains that preferentially expose the glycosidic bond to nucleophilic attacks or β-elimination reactions. Indeed, these linear regions behave more like pure mannan, which has been reported to be less stable in basic conditions compared to galactomannans [36]. However, we underline that such polymer–polymer interactions act together with the above-mentioned factors rather than being the sole cause of the observed rheological response. In contrast, the xanthan-based hydrogels responded positively to the incorporation of CC. Regardless of the NaOH treatment, both G′ and G″ slightly increased compared to the pure xanthan system, and all samples maintained strong gel properties (Figure 6c). Among the composite samples, the viscoelastic strength followed the trend HX_CC_0.04_ > HX_CC_10_ > HX_CC_5_, indicating that the alkaline pretreatment influenced the gel structure to some extent. However, no clear monotonic relationship emerged between NaOH concentration and gel enhancement; instead, a U-shaped trend was observed, with the highest values corresponding to the lowest alkaline treatment and the lowest ones to the intermediate NaOH concentration. This suggests that xanthan’s interaction with CC is governed primarily by specific intermolecular associations rather than being tuned by the alkalinity of the preparation medium.

These findings underscore the importance of the polysaccharide matrix type in determining the structural response to cellulose-based additives. While xanthan benefits from cellulose incorporation, guar and tara exhibit destabilization that may limit their use in composite gel systems requiring high mechanical integrity.

The temperature-dependent viscoelastic behavior of the hydrogel mixtures was further investigated through small-amplitude oscillatory measurements over the range from room temperature to 75 °C (Figure S6). Frequency sweeps were recorded every 10 °C to monitor the evolution of the mechanical response with temperature. Figure S6 reports the highest temperatures at which each system maintains gel characteristics before the transition to a liquid-like state. At elevated temperatures, both HG_CC and HX_CC composite hydrogels exhibit a progressive decrease in viscoelastic strength. As shown in Figure S4a,b, the storage modulus (G′) of the guar-based system remains dominant and nearly frequency-independent up to 55 °C, beyond which G′ and G″ invert, indicating gel breakdown and a transition to a viscous liquid. In contrast, the xanthan-based composites preserve their gel character up to 65 °C, confirming the higher thermal stability of the xanthan–CC network. The magnitude of G′ in the gel phase is influenced by the alkaline treatment applied to cellulose citrate: for the guar system, sample HG_CC_0.04_ exhibits the highest modulus, while HG_CC_10_ and HG_CC_5_ show progressively weaker gel characteristics. Conversely, tara-based hydrogels do not form a gel under any of the tested conditions; G″ remains higher than G′ across all temperatures, confirming their viscous liquid nature. Nonetheless, the sequence of alkaline treatments still affects rheology, following the same destabilizing trend (HT_CC_0.04_ < HT_CC_10_ < HT_CC_5_), with higher NaOH concentrations further impairing network formation.

Overall, the rheological analysis demonstrates that thermal resistance and structural integrity strongly depend on the polysaccharide-cellulose compatibility and on the alkalinity of cellulose citrate preparation. Xanthan-based systems show superior gel stability and temperature tolerance, while guar and tara matrices are more prone to thermal and compositional destabilization.

##### Quantitative Rheological Evaluation Using the Weak Gel Model

Further rheological analysis was carried out on the prepared samples using the Weak Gel model, which enables the extraction of quantitative parameters from frequency sweep data, specifically, the interaction strength (a) and the coordination number (z) (Table S1) [37]. These parameters provide insight into, respectively, the intensity and connectivity of the interactions within the hydrogel network.

**Figure 7:**
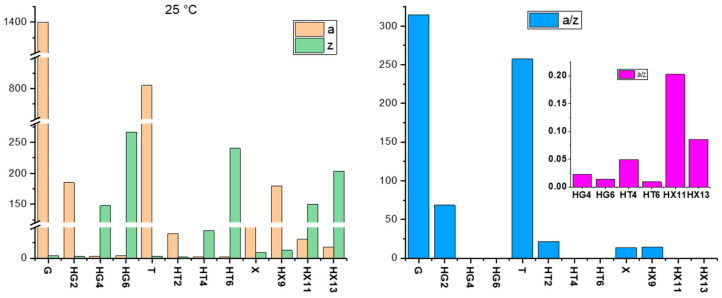
Values of the parameters a and z extracted from the weak gel model for pure and composite hydrogels at 25 °C.

At 25 °C, the pure gum hydrogels exhibit markedly different interaction strengths, with a(G) ≈ 1400, a(T) ≈ 800, a(X) ≈ 150, confirming that the HG_0_ is the most cohesive network, while Xanthan presents the weakest individual interactions. Upon incorporation of cellulose citrate, the interaction strength (a) decreases whereas the coordination number (z) increases in all systems (Figure 7). This trend indicates that the introduction of CC weakens the individual crosslinking interactions but promotes a higher degree of structural connectivity within the network. When the interaction strength decreases while the coordination number increases, this implies that the individual crosslinks within the network become less rigid, but the overall number of connections grows, leading to a more extended and cooperative structure.

In this context, CC acts as a network reorganizing agent, reducing the strength of direct gum–gum interactions while simultaneously introducing new contact points or multiple linkage sites (i.e., an increase in the number of rheological units correlated to one another in the 3D structure) through its flexible chains and pendant carboxylate groups. The result is a redistribution of the interactions within the hydrogel, where local rigidity is reduced but the network as a whole becomes more cohesive and structurally integrated.

The decrease in the a/z ratio thus indicates a shift from networks governed by a limited number of strong junctions to architectures defined by many weaker but cooperative interactions, in line with the development of a more flexible and distributed polymer–cellulose structure. This behavior, however, manifests differently among the three systems: in Guar- and Tara-based hydrogels, the weakening of individual interactions predominates, leading to a partial loss of structural integrity and a softer network; conversely, the Xanthan-based hydrogels benefit from this rearrangement, as the increased coordination compensates for the reduction in interaction strength, resulting in a more homogeneous and cooperative network that enhances the gel’s overall stability. This is probably due to the carboxylic acid groups present on the glucuronic and pyruvylated Mannose units of XG, that compatibly interact and complement the pendant carboxylic acid groups of CC.

It is also noteworthy that the influence of the different alkaline treatments follows a similar trend: increasing alkalinity tends to lower the interaction strength in Guar and Tara systems, while in Xanthan the effect is more complex, depending on both the cellulose content and the NaOH concentration. Overall, these results confirm that the polysaccharide matrix plays a decisive role in dictating the network architecture, and that the Xanthan–cellulose citrate system uniquely benefits from the synergistic assembly of the two components. Importantly, the overall trend is maintained with increasing temperature, albeit with a general decrease in absolute values due to kinetic effects, highlighting a good buffering capacity of the systems against thermal perturbations (Figure S7 and Table S1).

##### Crosslinking Density (ν_e_) of Natural Gum-Based Hydrogels and the Effect of Citrated Cellulose Incorporation

Hydrogels prepared from pure natural gums exhibit markedly different apparent crosslinking densities (*ν*_*e*_) depending on the polysaccharide type. Guar gum forms the most compact and rigid network (*ν*_*e*_ = 323.8 mol m^−3^), followed by tara gum (143.7 mol m^−3^), whereas xanthan gum displays the lowest value (37.08 mol m^−3^), consistent with a more open and deformable network structure. These variations reflect the distinct molecular organization and intrinsic ability of each polymer to establish intermolecular junctions, which ultimately control the mechanical strength of the gels.

The incorporation of CC markedly affects these parameters, with the outcome strongly depending on the type of gum matrix. In guar-based hydrogels (HG_CC_10_, HG_CC_5_, HG_CC_0.04_), the apparent crosslinking density sharply decreases from 323.8 to 26.7–53.5 mol m^−3^, indicating a transition toward a softer, less compact structure. An even more pronounced reduction is observed in tara-based systems (HT_CC_10_, HT_CC_5_, HT_CC_0.04_), where *ν*_*e*_ values drop to 2.7–21.9 mol m^−3^, suggesting a significant disruption of the pre-existing network architecture. In contrast, xanthan-based hydrogels (HX_CC_10_, HX_CC_5_, HX_CC_0.04_) exhibit a clear strengthening effect, with *ν*_*e*_ rising from 37.08 to 70.96 mol m^−3^, revealing enhanced crosslink formation.

**Figure 8:**
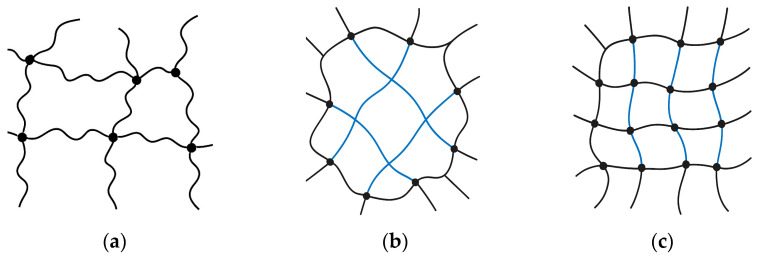
Schematic representation of (**a**) hydrogel polymer network; (**b**) effect of cellulose citrate in guar and tara hydrogels, where it disrupts part of the network connections, resulting in fewer crosslinking points and a more open structure; (**c**) effect of cellulose citrate in xanthan hydrogels, where it forms additional connections, leading to more crosslinking points and a denser network.

This opposite behavior can be rationalized in terms of molecular compatibility and interaction mechanisms. In guar and tara, the bulky, highly hydrophilic CC chains interfere with native interchain associations, leading to an expansion of the network mesh and a reduction in effective junction density. Conversely, in xanthan, the polyanionic backbone and the presence of ordered helical domains facilitate additional hydrogen bonding and possible ionic interactions with CC, resulting in the formation of supplementary crosslinking sites and a more compact, reinforced structure (Figure 8).

Overall, these results demonstrate that cellulose citrate can act either as a network-reinforcing or network-loosening agent, depending on the structural and chemical characteristics of the polysaccharide matrix, highlighting the crucial role of polymer–polymer compatibility in determining the final architecture of composite hydrogels.

This behavior highlights the dual role of the citrate moieties in the gel formation process. Under alkaline conditions, partial hydrolysis of the citrate ester groups may occur, releasing free citrate species into the medium. These negatively charged groups can interact through hydrogen bonding and electrostatic bridges with hydroxyl or carboxyl sites mediated by the sodium ions and water, on the polysaccharide chains, thereby stabilizing the gel structure despite the partial degradation of the cellulose backbone. This mechanism explains why, even in systems where network weakening is observed (such as guar and tara), a coherent gel phase is still maintained, and why xanthan, rich in carboxyl functionalities, forms the most reinforced composite gels at higher NaOH concentrations.

## 3. Conclusions

This study investigated natural gum-based hydrogels (HG, HT, HX) and their composites with cellulose citrate (CC), focusing on the influence of CC incorporation and alkaline pretreatment on structural, thermal, and rheological properties. All natural gums formed stable hydrogels, though with distinct viscoelastic behaviors reflecting their molecular architectures.

The addition of CC markedly affected gel characteristics: while guar- and tara-based systems showed network weakening and reduced crosslinking density, XG–CC composites exhibited enhanced mechanical integrity and thermal stability. This reinforcement arises from synergistic hydrogen bonding and ionic interactions between carboxylate groups of xanthan and cellulose citrate.

Thermal and rheological analyses confirmed that alkaline pretreatment of CC modulates the gel network by altering the degree of polymer and citrate hydrolysis and the distribution of water–polymer interactions. The Weak Gel model analysis further revealed that CC acts as a network reorganizer, increasing structural connectivity while softening local junctions.

The structural, thermal, and rheological behaviors observed in our hydrogels are consistent with those reported for related polysaccharide- and cellulose-based systems [38,39]. The FT-IR features, including the broad OH bending band at 1650–1700 cm^−1^ and the weak ester carbonyl signal of cellulose citrate near 1736 cm^−1^, align with spectra commonly described for hydrated polysaccharide networks and citric acid-modified cellulose. Likewise, the two-step thermal degradation of the pure gums and the multi-step profile of cellulose citrate match previous TGA studies on natural-polymer hydrogels [40]. Rheologically, the strong-gel character of the neat systems (G′ ≫ G″) falls within the typical range for guar, tara, and xanthan gels reported in the literature. The opposite effects of CC incorporation, network weakening in guar and tara, but reinforcement in xanthan, also agree with known differences in gum compatibility and intermolecular interactions [9,41]. These comparisons confirm that the behavior of our systems is consistent with that of established natural-gum hydrogels, while highlighting the particularly robust performance of xanthan–cellulose citrate composites for soft-material applications.

Overall, combining natural gums with cellulose citrate provides a sustainable route to design biopolymeric hydrogels with tunable viscoelastic and thermal properties. In particular, xanthan–cellulose citrate hydrogels demonstrate the most promising balance of flexibility, stability, and responsiveness, making them attractive candidates for optoelectronics, wearable and skin-interfaced devices, such as soft sensors or stretchable energy-storage components. The application of these hydrogels may be limited by their sensitivity to dehydration or environmental fluctuations, potentially impacting their operational reliability in wearable or skin-interfaced devices. This aspect is currently being evaluated in our ongoing device-level studies, which will further clarify the stability requirements and guide future optimization of these sustainable soft materials.

## 4. Materials and Methods

### 4.1. Materials

Tara, guar, and xanthan gums were provided by JRS Silvateam Food Ingredients (Rende (CS), Italy). Cellulose citrate (CC) was synthesized according to the procedure of Romeo and coworkers [17].

### 4.2. Hydrogels Preparation

Hydrogels based on natural gums (HG, HT, and HX) and composite hydrogels combining natural gums with CC (HG_CC, HT_CC, and HX_CC series) (Table 1) were synthesized via a straightforward procedure under mild conditions. Pure gum hydrogels were prepared by dispersing the corresponding gum powders in 100 mL of ultrapure water, previously heated and maintained at 90 °C and the mixtures were stirred for 20 min. For the preparation of composite hydrogels, three different aqueous media were used to incorporate CC:

- 10% NaOH solution: to completely dissolve CC.
- 5% NaOH solution: to prepare a CC suspension.
- Ultrapure water containing 0.04% NaOH: in this case CC was first dispersed and sonicated to improve dispersion. The minimal amount of base slightly increased the pH, but was necessary to inhibit microbial growth during gelation.

**Table 1:** Composition of hydrogel samples.

Gum Type	Marked Name	Gum (*w*/*w*)%	Cellulose Citrate (*w*/*w*)%	NaOH (*w*/*w*)%	^a^ Gel/Liquid Phase Transition Temperature T_G/L_ (°C)
Guar	HG_0_		-	-	70–80 °C
	HG_CC_10_	2.25	0.075	10	70
	HG_CC_5_			5	70
	HG_CC_0.04_			0.04	70
Tara	HT_0_		-	-	60–70 °C
	HT_CC_10_	2.25	0.075	10	50
	HT_CC_5_			5	50
	HT_CC_0.04_			0.04	50
Xanthan	HX_0_		-	-	80 °C
	HX_CC_10_	2.25	0.075	10	80 °C
	HX_CC_5_			5	80 °C
	HX_CC_0.04_			0.04	80 °C

^a^ Determined by the test-tube inversion method.

In all cases, the CC solution or suspension was gradually added to the gum. To ensure a valid comparison of the modifications induced by incorporating CC into the three natural gums, the optimal CC–Gum–water weight ratio was first determined. A ratio of 0.075:2.25:100, corresponding to a 3.3% of CC with respect to the polymer gum was selected as the maximum amount of CC that can be employed to avoid any significant transmittance reduction of the gel. Indeed, for higher levels of CC, the gels became darker in color (see Figure S8), which is undesirable for optoelectronic applications. Figure S8 shows the transmittance (T%) of xanthan hydrogel films, as a function of the amount of CC, across the whole visible range (380–800 nm). It can be observed that there is a significant reduction in the film transmittance (~11%) going from 3.3% of CC to 5.5%, already at a gel thickness as small as 15 μm. A summary of the samples that will be thoroughly discussed in this work is reported in Table 1.

### 4.3. Characterization of Hydrogels

#### 4.3.1. Fourier Transform Infrared Spectroscopy (FT-IR)

Fourier Transform Infrared (FT-IR) analysis was carried out on the hydrogel samples in the mid-infrared area (4000–400 cm^−1^) with a Perkin Elmer Spectrum 100 FT-IR spectrometer (PerkinElmer, Shelton, CT, USA). The hydrogels were placed in the dedicated sample holder designed for soft and hydrated materials, ensuring proper contact with the IR beam without altering their hydration state. Each spectrum was collected by averaging 64 scans at a resolution of 4 cm^−1^, and a background spectrum was recorded prior to each measurement. The spectra were subsequently baseline-corrected and normalized to allow comparison among samples. All measurements were carried out in triplicate to ensure reproducibility, and were performed at room temperature.

#### 4.3.2. Optical Transmittance Measurements

The optical transmittance of the hydrogel films was measured with a UV–Vis–NIR Jasco V-770 spectrophotometer (JASCO Corporation, Tokyo, Japan). Measurements were performed on ITO/hydrogel film/ITO cells mounted in the instrument’s sample holder, and the transmittance was recorded in the 380–800 nm range at room temperature against an empty cell with the same cell gap. The films were made by laminating the hydrogels between two indium tin oxide (ITO) glass slides. The hydrogel film thickness was set at 15 μm by utilizing specific spacers.

#### 4.3.3. Thermogravimetric Analysis (TGA)

TGA was performed on a Hitachi STA200RV thermogravimetric analyzer (Hitachi, Tokyo, Japan) (Real Vision, i.e., equipped with an internal webcam to observe the decomposition during the analysis). Approximately 20 mg of each sample was placed in an alumina crucible. The acquisition was carried out in an inert environment, under a flow of nitrogen at a constant speed of 100 mL/min, starting from room temperature with a heating of the sample at 10 °C/min up to a temperature of 580 °C. The DTG curves, which provide the percentage weight loss rate, were obtained from the first derivative of the TG profiles.

#### 4.3.4. Dynamic-Mechanical Analysis/Rheological

The rheological measurements were conducted using a shear stress-controlled rheometer DSR 5 (Rheometrics, Piscataway, NJ, USA) equipped with a parallel plate (diameter 25 mm) over the temperature range 25–80 °C. The temperature was controlled by a Peltier apparatus (±0.1 °C). For each gel, the temperature range over which a self-supporting gel phase (G) exists and the gel–liquid transition temperature (T_G/L_) were first determined using the test-tube inversion method. Test tubes containing the hydrogels were placed in a water bath and heated from room temperature until the samples began to flow, indicating formation of the liquid phase. The corresponding T_G/L_ values for all formulations are summarized in Table 1.

To prevent errors due to evaporation, the measuring geometries were surrounded by a low viscous silicone oil. In particular a silicone oil of viscosity of 0.001 Pa.s was wrapped around the edge to prevent water evaporation. The fresh samples that were submitted to experiments were fully transparent and free from air bubbles. Dynamic shear experiments were performed in a frequency range between 0.1 and 15.9 Hz within the linear viscoelastic region. The small-amplitude dynamic tests provided information on the linear viscoelastic behavior of materials through the determination of the complex shear modulus (Equation (1)) [42].

(1) $$G^{*}\left(\omega \right)=G^{\prime }\left(\omega \right)+iG^{\prime \prime }\left(\omega \right)$$

where $G^{\prime }\left(\omega \right)$ is the in phase (or storage) component and $G^{\prime \prime }\left(\omega \right)$ is the out-of-phase (or loss) component. $G^{\prime }\left(\omega \right)$ is a measure of the reversible, elastic energy, while $G^{\prime \prime }\left(\omega \right)$ represents the irreversible viscous dissipation of the mechanical energy. The applied strain amplitude for the viscoelastic measurements was reduced until the linear response regime was reached. This analysis was made by performing strain sweep tests in all temperature range investigated. Weak Gel Model [37] was also applied to some of the oscillatory spectra (Equation (2)):

(2) $$\left|G^{*}(\omega )\right|=\sqrt{G^{\prime }{\left(\omega \right)}^{2}+G^{\prime \prime }{\left(\omega \right)}^{2}}=a\omega^{1/z}$$

This model approaches the structural behavior of the samples following the physical idea of a weakly structured network where the mechanical properties are given by different rheological units connected to each other with a defined interaction force. This simple model is directly correlated with the parameters of Equation (1): “a” represents the strength of the interaction among the rheological units: a sort of intensity of cooperative interactions, and “z” corresponds to the number of rheological units interacting with each other and can be understood as coordination number. Combinations of the two parameters are able to estimate the evolution of mechanical properties in oscillatory kinematic [43], also as a function of temperature.

#### 4.3.5. Apparent Crosslinking Density Calculation

The apparent crosslinking density ($v_{e}$) of the hydrogels was determined from rheological measurements using the theory of rubber elasticity. The storage modulus ($G^{\prime }\left(\omega \right)$) was obtained in the linear viscoelastic region (LVR) from oscillatory shear measurements at a frequency of 0.15915 Hz and a temperature of T = 25 °C. The strain amplitude was selected within the LVR as determined from strain sweep tests. The crosslinking density was calculated according to Equation (3):

(3) $$v_{e}=\frac{G^{\prime }}{RT}$$

where R is the universal gas constant (8.314 J mol^−1^ K^−1^), T is the absolute temperature, and $G^{\prime }\left(\omega \right)$ is the storage modulus in the plateau region. $G^{\prime }\left(\omega \right)$ values are in Pascal.

## Data Availability

The original contributions presented in this study are included in the article/Supplementary Materials. Further inquiries can be directed to the corresponding authors.

## Supplementary Materials

The following supporting information can be downloaded at: https://www.mdpi.com/article/10.3390/gels11121005/s1, Figure S1: Comparison of FT-IR spectra of pure natural gum hydrogels (HG_0_, HT_0_, HX_0_) and composite hydrogels (HG_CC, HT_CC, HX_CC); Figure S2: FT-IR spectra of the cellulose citrate powder; Figure S3: TGA and DTG curves of Cellulose citrate powder; Figure S4: Derivative thermogravimetric curves (DTG) of pure and composite hydrogels, zoomed in the 30–150 °C range; Figure S5: Dynamic moduli (G′ and G″) of pure gum hydrogels at 25 °C; Figure S6: Comparison of the dynamic storage modulus (G′) for (a) HG_0_ at 25 °C and composite HG_CC hydrogels at 55 °C, and (b) HX_0_ at 25 °C and composite HX_CC hydrogels at 65 °C; and of the storage (G′) (c) and loss (G″) (d) moduli for HT_0_ at 25 °C and composite HT_CC hydrogels at 45 °C, as a function of NaOH treatment; Figure S7: Values of the parameters a and z extracted from the weak gel model for pure and composites hydrogels at 45 °C; Figure S8: Transmittance of xanthan hydrogel films (15 μm thickness) as a function of the cellulose citrate percentage. The inset shows images of the films where the gel coloration difference among the samples can be appreciated; Table S1: Table of parameters a and z for the analyzed samples at each temperature.
