# Supplementary material for: Eco-Friendly Hydrogels from Natural Gums and Cellulose Citrate: Formulations and Properties
Source: Gels. 2025 Dec 12;11(12):1005. doi: 10.3390/gels11121005 (PMC12733339; doi:10.3390/gels11121005)
Supplement: Supplementary file 1 [file gels-11-01005-s001.zip › gels-4007726-supplementary.pdf]

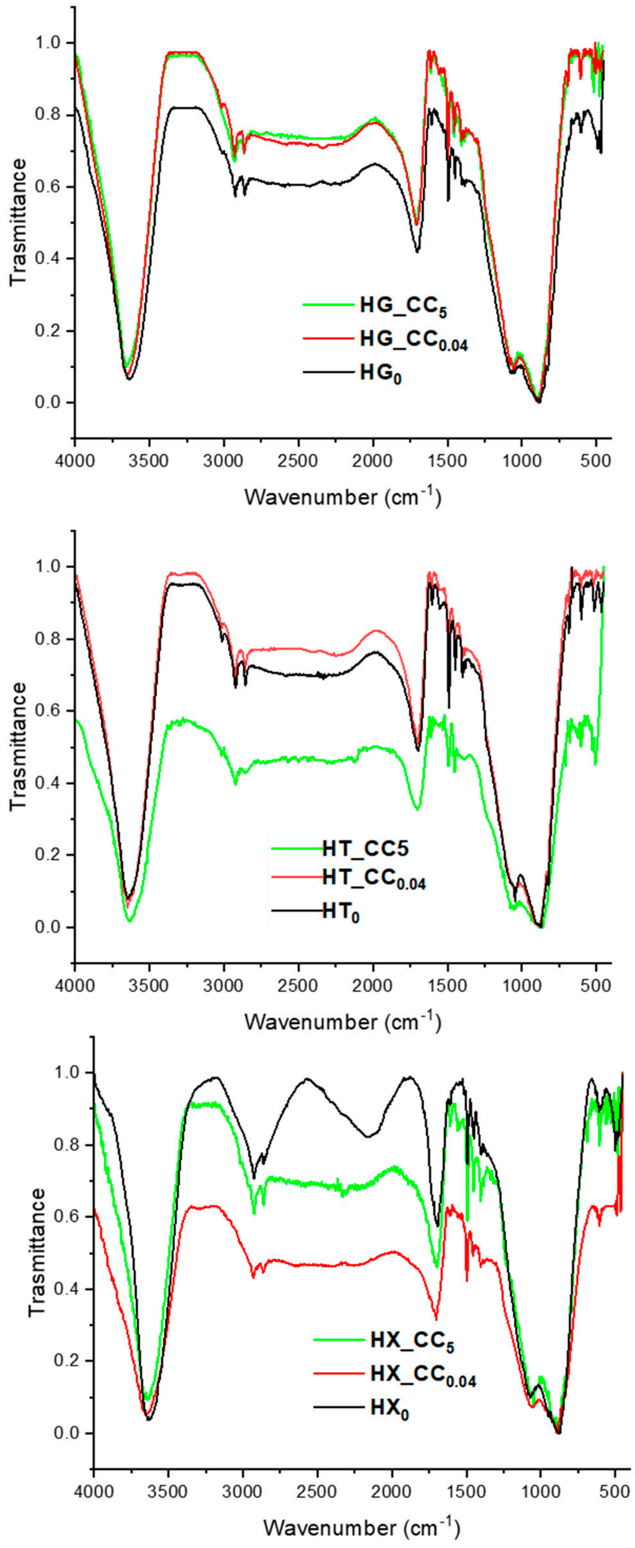

Figure S1. Comparison of FT-IR spectra of pure natural gum hydrogels (HG_0, HT_0, HX_0) and composite hydrogels (HG_CC, HT_CC, HX_CC).

FT-IR spectrum of CC

The FT-IR spectrum of CC displays all the characteristic absorption bands of the cellulose backbone, including a broad O–H stretching at 3300–3400 cm^{-1}, C–H stretching near 2900 cm^{-1}, O–H bending of bound water around 1640 cm^{-1}, CH_2 bending at ~1430 cm^{-1}, C–O–C asymmetric stretching at ~1160 cm^{-1}, and C–O stretching in the 1050–1060 cm^{-1} region. In addition to these features, a strong absorption band is observed at 1744 cm^{-1}, attributed to the C=O stretching vibration of ester and carboxylic acid groups. The overall spectrum indicates that the solid product retains the cellulose backbone as its main structural component while incorporating ester functionalities.

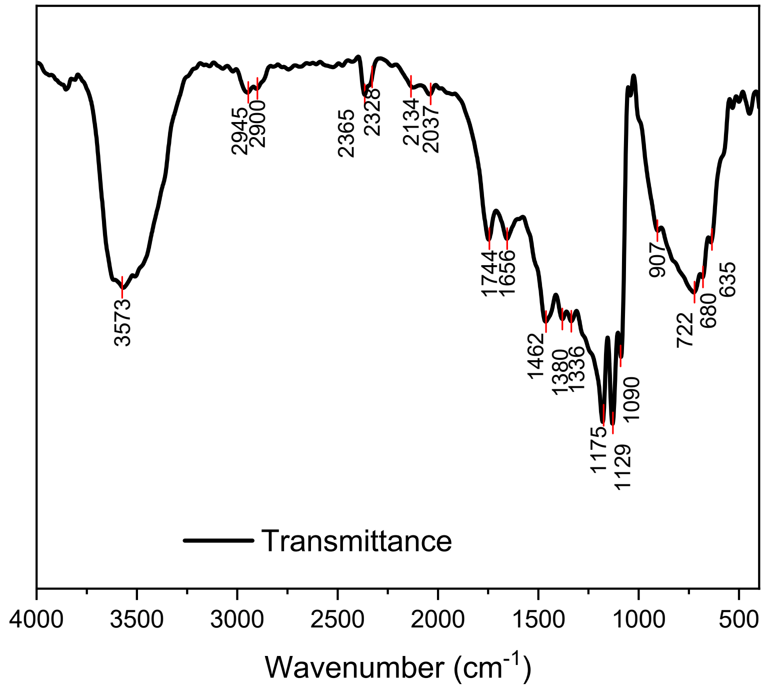

Figure S2. FT-IR spectra of the cellulose citrate powder.

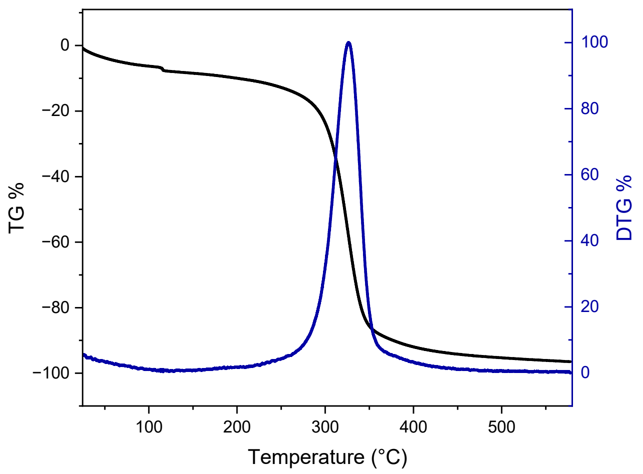

Figure S3. TGA and DTG curves of Cellulose citrate powder.

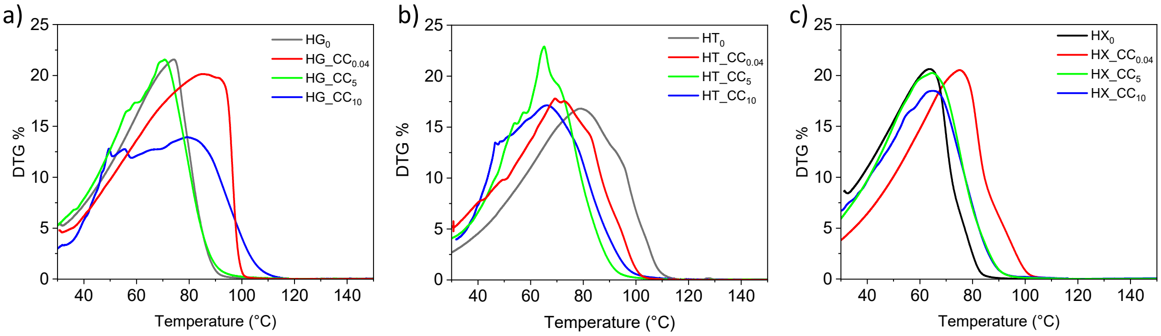

Figure S4. Derivative thermogravimetric curves (DTG) of pure and composite hydrogels, zoomed in the 30–150 °C range.

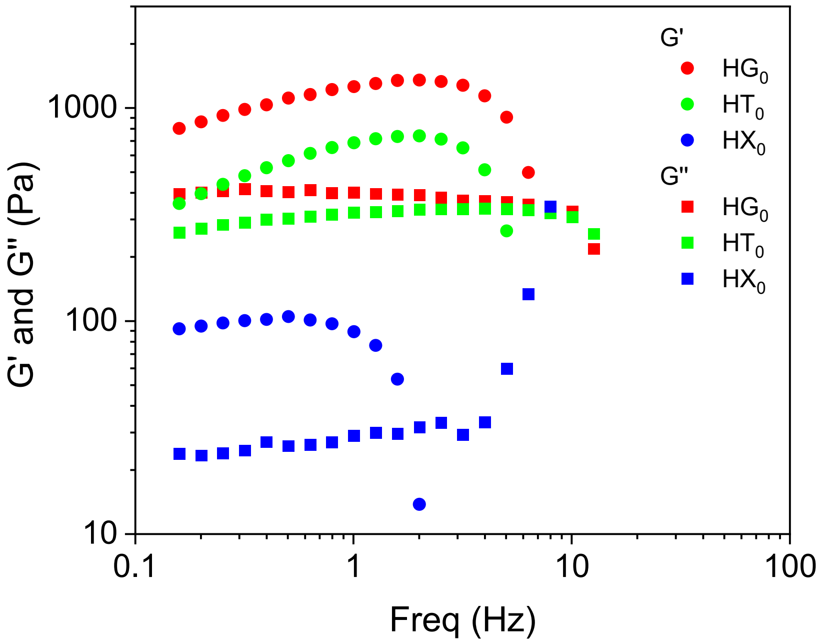

Figure S5. Dynamic moduli (G′ and G″) of pure gum hydrogels at 25 °C.

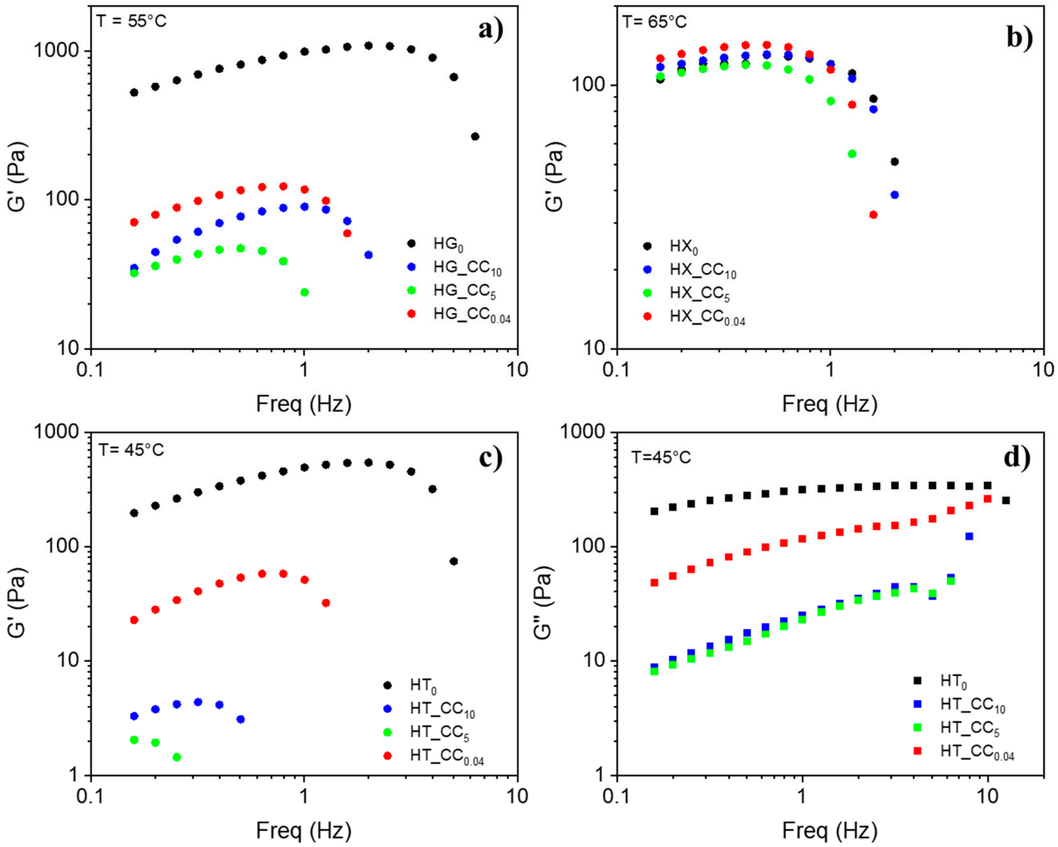

Figure S6. Comparison of the dynamic storage modulus (G′) for (a) HG_0 at 25 °C and composite HG_CC hydrogels at 55 °C, and (b) HX_0 at 25 °C and composite HX_CC hydrogels at 65 °C; and of the storage (G′) (c) and loss (G″) (d) moduli for HT_0 at 25 °C and composite HT_CC hydrogels at 45 °C, as a function of NaOH treatment.

Table S1. Table of parameters a and z for the analyzed samples at each temperature.

Sample	T = 15 °C		T = 25 °C			T = 35 °C		T = 45 °C			T = 55 °C		T = 65 °C		T = 75 °C	
	a	z	a	z	a/z	a	z	a	z	a/z	a	z	a	z	a	z
HG$_0$	1300	5.7	1400	4.5	315	1300	3.9	1200	3.7	325	1120	3.3	1020	3.1	915	3
HG_CC$_{10}$	200	3.1	185	2.7	69	175	2.5	155	2.5	61	160	1.8				
HG_CC$_5$	3	180.3	3.4	147.7	0.02	3	121.6	4	98.8	0.04	3	86.6	3	79.5	3	54.81
HG_CC$_{0.04}$	4	298	3.8	266.6	0.01	4	237.8	3	211	0.02	3	185.8	3	158.6	3	128.15
HT$_0$	830	3.8	805	3.1	257	730	2.6	620	2.4	254	530	2.2	1000	2.2	350	2.25
HT_CC$_{10}$	40	2.2	40	1.8	22	35	1.9	25	1.9	13						
HT_CC$_5$	2	60.1	2.2	44.6	0.05	2	33.8	2	25.2	0.06	1	23.5	1	26.2	1	33.08
HT_CC$_{0.04}$	2	304.8	2.2	240.4	0.01	2	192.2	2	144.7	0.01	2	102.1	2	64.3	2	64.41
HX$_0$	130	4.7	120	8.7	14	110	14.7	120	8.3	14	115	14.1	140	9.6	130	6.49
HX_CC$_{10}$	180	15.4	180	12.7	14	175	12.5	165	14.1	12	155	13.4	140	12.7		
HX_CC$_5$	34	152.2	30.4	150.2	0.2	24	145.9	38	138.8	0.3	13	141	19	127.7	11	125.31
HX_CC$_{0.04}$	35	206.7	17.5	203.4	0.9	17	192.5	15	179.9	0.08	14	168.6	13	155.1	7	91.71

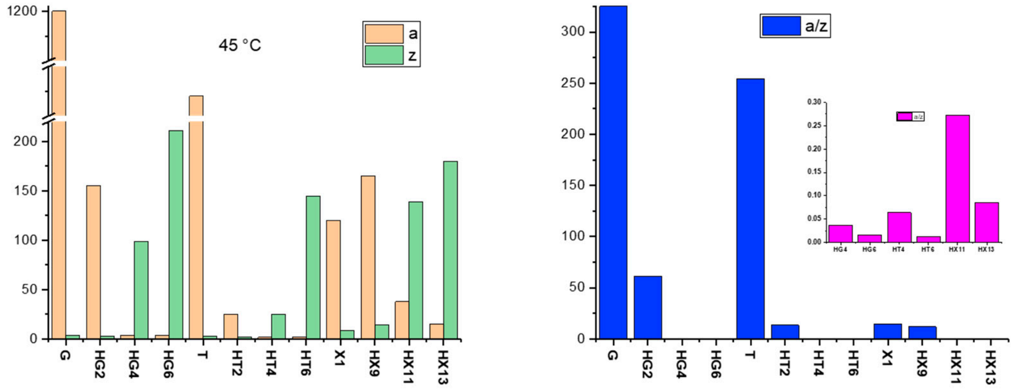

Figure S7. Values of the parameters a and z extracted from the weak gel model for pure and composites hydrogels at 45 °C.

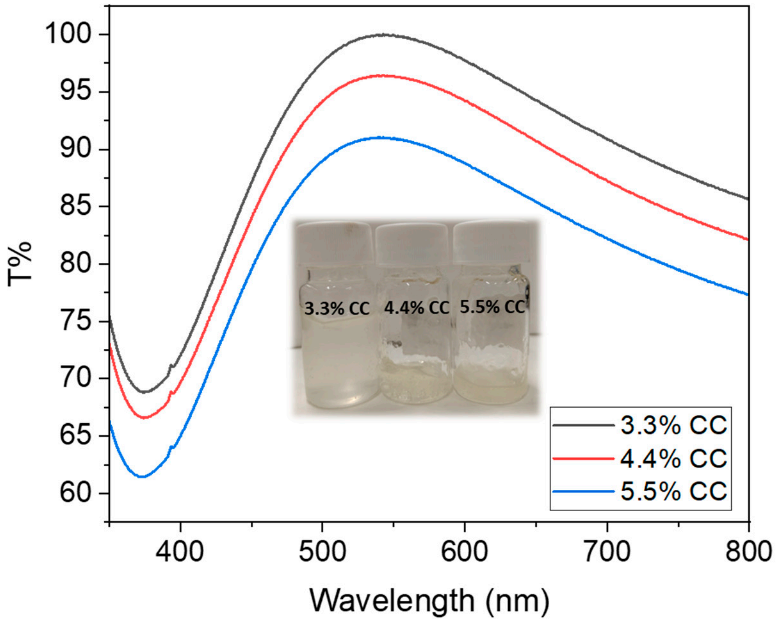

Figure S8. Transmittance of xanthan hydrogel films (15 **µ**m thickness) as a function of the cellulose citrate percentage. The inset shows images of the films where the gel coloration difference among the samples can be appreciated.
